# Novel Expression of *GLUT3*, *GLUT6* and *GLUT10* in Equine Gluteal Muscle Following Glycogen-Depleting Exercise: Impact of Dietary Starch and Fat

**DOI:** 10.3390/metabo13060718

**Published:** 2023-06-01

**Authors:** Stephanie J. Valberg, Deborah Velez-Irizarry, Zoe J. Williams, Joe D. Pagan, Vanesa Mesquita, Brian Waldridge, Hailey Maresca-Fichter

**Affiliations:** 1 McPhail Equine Performance Center, Department of Large Animal Clinical Sciences, College of Veterinary Medicine, Michigan State University, 736 Wilson RD, East Lansing, MI 48824, USA; 2 Kentucky Equine Research, 3910 Delany Ferry Rd., Versailles, KY 40383, USA

**Keywords:** glucose transport, horse, nutrition, carbohydrates, training

## Abstract

Horses have a slow rate of muscle glycogen repletion relative to other species for unknown reasons. Our aim was to determine the expression of glucose transporters (*GLUT*) and genes impacting GLUT4 expression and translocation in the gluteal muscle. Five fit Thoroughbred horses performed glycogen-depleting exercises on high-starch (HS, 2869 g starch/day) and low-starch, high-fat diets (LS-HF, 358 g starch/d) with gluteal muscle biopsies obtained before and after depletion and during repletion. Muscle glycogen declined by ≈30% on both diets with little increase during repletion on LS-HF. Transcriptomic analysis identified differential expression (DE) of only 2/12 genes impacting GLUT4 translocation (two subunits of AMP protein kinase) and only at depletion on LS-HF. Only 1/13 genes encoding proteins that promote *GLUT4* transcription had increased DE (*PPARGC1A* at depletion LS-HF). *GLUT4* comprised ≈30% of total *GLUT* mRNA expression at rest. Remarkably, by 72 h of repletion expression of *GLUT3*, *GLUT6* and *GLUT10* increased to ≈25% of total *GLUT* mRNA. Expression of *GLUT6* and *GLUT10* lagged from 24 h of repletion on HS to 72 h on LS-HF. Lacking an increase in *GLUT4* gene expression in response to glycogen-depleting exercise, equine muscle increases *GLUT3*, *GLUT6* and *GLUT10* expression potentially to enhance glucose transport, resembling responses observed in resistance trained GLUT4-null mice.

## 1. Introduction

Glycogen, a highly branched glucose polymer, is the primary intramuscular fuel for aerobic and anaerobic exercise across species. Within 24 h of intense exercise in humans, muscle glycogen is restored to concentrations of 1–1.5% (g glycogen/100 g muscle wet weight muscle) [1]. This is achieved by rapid translocation of glucose transporter 4 (GLUT4) to the sarcolemma from intracellular membrane pools and increased activity of glycogen synthase [2]. Super-compensatory increases in muscle glycogen of 2 to 4-fold can be achieved in humans by consuming a high-starch meal after intense exercise to enhance insulin-stimulated GLUT4 translocation [3]. After physiological insulin stimulation, in vitro GLUT4 translocation increases by 80%, in humans and by 400% after supraphysiological insulin stimulation [4]. Exercise also enhances GLUT4 gene and protein expression in humans [5,6,7]. 

In contrast to humans, in horses, it often takes up to 72 h to restore muscle glycogen to pre-exercise concentrations of 1.5–2% (g glycogen/100g muscle wet weight) after glycogen-depleting exercise [8,9,10]. Low-starch (180–250 g/horse/day), compared to high-starch, diets (2800–3500 g/day) further delay glycogen resynthesis in horses [10]. Muscle glycogen supercompensation cannot be achieved in horses, either by feeding high-starch meals (corn), oral glucose (22 g/kg body weight) or by providing IV glucose infusions (6 g glucose/kg body weight for 12 h) following exercise [10,11,12]. Although GLUT4 is expressed in equine muscle, in vitro insulin stimulation at physiological levels only increases GLUT4 translocation by 15%, and neither hyperinsulinemia nor exercise increase muscle GLUT4 content [13,14]. The mechanism behind limitations to exercise- and insulin-stimulated muscle glucose transport and glycogen resynthesis in the horse remains unknown. 

GLUT4 is one of 12 facilitative glucose transporters encoded by the gene family *SLC2A* [15]. The terms *GLUT1* through *GLUT12* are often used by convention for the corresponding *SLC2A1* through *SLC2A12* genes [16]. GLUT1, GLUT3, GLUT4, GLUT5, GLUT10 and GLUT12 are known to be expressed in human skeletal muscle [15], whereas GLUT1, GLUT4, GLUT8 and GLUT12 are known to be expressed in equine muscle [14,17,18]. 

We hypothesized that expression of *GLUT4* and genes that impact GLUT4 expression and translocation would not change during glycogen depletion and repletion in equine muscle. Further we hypothesized that transcriptomic analysis of equine muscle would identify novel glucose transporters expressed during glycogen repletion. We investigated gene expression when horses were fed a high-starch (HS) diet as well as an isocaloric, isonitrogenous, low-starch, high-fat (LS-HF) diet. The first objective of our study was to investigate the expression of genes that encode regulators of GLUT4 translocation and *GLUT4* transcription after glycogen-depleting exercise and during repletion, comparing expression between depletion and repletion time points with pre-exercise expression. The second objective was to determine temporal differential expression (DE) of facilitative glucose transporters (*GLUT1* through *GLUT12)*, comparing depletion and repletion time points to pre-exercise expression in horses fed HS and LS-HF diets. The third objective of our study was to determine the proportionate expression of all genes encoding facilitative glucose transporters in equine gluteal muscle at rest, following glycogen-depleting exercise and during repletion, comparing expression in horses fed the HS to expression on the LS-HF diet. 

## 2. Materials and Methods

This study utilized muscle samples that had been obtained during a 2012 diet and exercise trial and were subsequently stored at −80 °C. The original study was designed to evaluate the effect of three isocaloric, isonitrogenous diets, a high-starch diet (HS, 2869 g/d), a medium-starch diet (MS, 2007 g/d) and a low-starch, high-fat (LS-HF, 358 g/d) diet on exercise performance and the rate of glycogen repletion in the gluteus medius muscle of Thoroughbred horses. For the present transcriptomic study, muscle biopsy samples from horses fed the HS and LS-HF diets, and not the MS diet, were utilized because of the cost of analyses. Because the original randomized Latin square design used three diets, the entire experimental design is described below.

### 2.1. Experimental Design

Six fit Thoroughbred geldings aged 8.2 ± 2 yrs., 555 ± 29.9 kg BW, Body condition scores 6–6.5 were studied in a 3 × 3 replicated Latin Square design. Prior to beginning the study, there was a three-week training period. The horses exercised 6 days per week on either the treadmill or mechanical walker. One day per week, the horses were not exercised and were turned out in small paddocks for 4–6 h while wearing muzzles that prevented grazing. During each 1-month study period, horses were trained for 3 weeks, alternating treadmill exercise and a mechanical walker, with one day off per week on pasture while wearing a grazing muzzle. During the fourth week, horses performed a 3-day exercise protocol on the treadmill at a 3° incline. The protocol consisted of an incremental exercise test on day one, followed by two days of intense interval exercise. During the one-week wash-out period between diets, the horses were exercised daily on the mechanical walker for approximately 30 min and were fed timothy hay, electrolytes, a vitamin/mineral supplement and a textured feed that contained 45% oats, 45% cracked corn and 10% molasses.

On the day of the incremental exercise test, a 14-gauge 13 cm catheter (MILA International Inc., Erlanger, Kentucky) was placed in the jugular vein. The incremental exercise test consisted of a five min warm-up at the walk (1.7 m/s), followed by two min at 4, 6 and 8 m/s and 1 min at 9, 10, 11, 12 and 13 m/s. At the end of the last gallop, the treadmill was immediately brought to 0° incline and horses were cooled off at the walk for five min. Blood samples (*n* = 11) were collected before exercise, during the final 15 s of each speed. Samples were centrifuged and plasma was stored at −20 °C until analyzed. 

**Figure 1:**
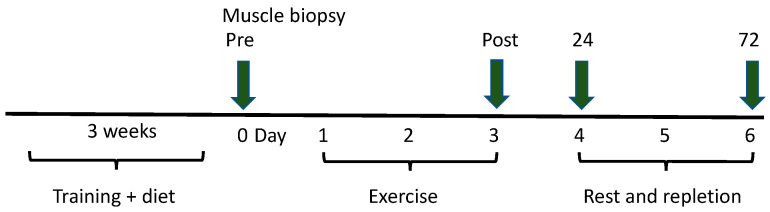
The design of the replicated 3 × 3 Latin square diet trial in which 6 horses were trained for three weeks on one of three diets. One horse did not complete the trial and was not included in statistical analyses. Gluteus medius muscle biopsies were obtained at rest and horses underwent three days of glycogen-depleting exercise with muscle biopsies taken immediately after the third exercise session and 24 and 72 h after cessation of exercise when horses were rested and fed their respective diets. After a washout period, horses were then switched to their assigned alternate diet and the protocol was repeated.

On days two and three of the depletion protocol, horses performed an interval-training workout consisting of 5 min at the walk (1.7 m/s) followed by 4 series of 5 min of trot (4 m/s) alternated with 2 min of gallop (10 m/s), with a cooling-off period of 5 min of trot (4 m/s) and 5 min of walk (1.7 m/s). The glycogen-repletion protocol consisted of 72 h of rest. Horses were fed their respective diets during repletion. Muscle samples were obtained one day prior to commencement of the depletion protocol and then immediately after completion of the third day of depleting exercise and at 24 and 72 h after the last exercise session (Figure 1). There was a 1-week washout period between diets during which horses were lightly trained similar to the pre-study training period. 

The protocol and procedures used in this study were performed in accordance with the Guide for Care and Use of Agricultural Animals in Agricultural Research and Teaching (Anon 1988). An exemption for the use of archived samples was obtained from the Institutional Animal Use and Care Committee at Michigan State University on 30 August 2022. 

### 2.2. Diet Composition and Intake

The glycemic index of the HS, MS and LS-HF concentrates was determined prior to the trial in 4 Thoroughbred horses that were fed 1 kg of the 3 concentrates described below, as well as 1 kg oats in a 4 × 4 Latin Square design. Blood samples were collected before and at 0, 30, 60, 90, 120, 150, 180, 210 and 240 min after the meal for determination of glucose concentrations. Oats were used as the index feed; glycemic indexes were expressed as area under the curve: AUC_feed_/AUC_oats_.

The horses were fed 1.25% BW/d timothy hay and 1.0% BW/d of either HS, MS or LS-HF concentrate along with an electrolyte supplement (Endura-Max, Kentucky Equine Research, Inc., Versailles, KY, USA) and 150 g/day of a vitamin mineral supplement (Micro-Max, Kentucky Equine Research, Versailles, KY, USA). Concentrates and forage were submitted to Dairy One (Dairy One Inc., Ithaca, NY, USA) for nutrient analysis (Tables S1 and S2). The nutrient compositions of the total diets (concentrate + hay) are shown in Table S1. The HS, MS and LS-HF rations provided 45%, 36% and 18% of total digestible energy (DE) from NSC, respectively; 11%, 15% and 23% of total DE from fat, respectively; and 30%, 33% and 45% of total DE from fiber, respectively. Daily intake of starch averaged 2869, 2007, and 358 g/d of NSC and 3974, 3078, and 1355 g/d in the HS, MS, and LS-HF rations, respectively. Horses received 1 kg of grass hay in the morning, 2 kg in the afternoon and the remainder of their calculated 1.25% kg BW amount in the evening.

Concentrates were withheld on the day of the depletion protocol and horses only received 1 kg of hay before exercise. Concentrate was offered to the horses as soon as they cooled off after exercise. Body weights were measured weekly on a portable digital scale (Equimetrics Inc., Redfield, AR, USA).

### 2.3. Blood Glucose

Plasma glucose was determined electrochemically using a glucose analyzer (YSI 2300 Stat Plus, Yellow Springs Instruments, Yellow Springs, OH, USA). 

### 2.4. Muscle Biopsies

Horses were sedated with 5 mg detomidine IV (Dormosedan, Pfizer Animal Health, New York, NY, USA) if necessary, prior to biopsy of the middle gluteal muscle. The biopsy site consisted of a 2.4 cm square 17 cm along a line running from the most dorsal part of tuber coxae to the head of the tail. Alternating sides and areas within the square were used for each subsequent biopsy. Briefly, subcutaneous lidocaine (Lidocaine-HCl-2%, Phoenix Pharmaceutical, Burlingame, CA, USA) was injected, an incision was made in the skin and the 6 mm diameter biopsy needle was inserted to a depth of 60 mm to obtain ≈200 mg of muscle. Biopsy specimens were immediately frozen in liquid nitrogen and stored at −80 °C. 

### 2.5. Glycogen Assay

Frozen muscle specimens from pre- and post-exercise, and after 24 h and 72 h of repletion were analyzed for each diet. Samples were weighed and portions (2–4 mg) of muscle tissue were boiled for 2 h in 1 M HCl. Glycogen was assayed fluorometrically as glucose residues [19]. 

### 2.6. Transcriptomic Analysis

#### 2.6.1. RNA Extraction and Sequencing

Total muscle RNA was extracted from muscle biopsies from pre-exercise, depletion and 24- and 72-h repletion for HS and LS diets for a total of 40 samples (2 diets × 4 samples/horse × 5 horses) [20]. RNA extraction was performed with TRIzol reagent (Invitrogen Corp, Carlsbad, CA, USA) using 35 mg of muscle tissue and following the manufacturer’s protocol. The quality and quantity of extracted total RNA were determined using the Agilent 2100 Bioanalyzer (Agilent Technologies, Inc., Santa Clara, CA, USA). Samples with a RIN ≥ 7 were used for RNA sequencing. Sequencing was performed at the Michigan State University Research Technology Support Facility (RTSF) Genomics Core. All 40 libraries were prepared using the Illumina TruSeq Stranded mRNA Library Preparation Kit on a Perkin Elmer Sciclone G3 robot and sequenced on the Illumina HiSeq 4000 platform (2 × 150 bp, paired-end reads). A total of 144 sequence files (282 Gb) consisting of ~86 million short-reads per library were obtained. 

#### 2.6.2. Mapping and Assembling

Raw RNA sequence reads were first filtered for adapter sequences using Trimmomatic [21], followed by quality trimming using ConDeTri, where the first 6 bases at the 3′ end and low-quality reads were filtered out, retaining reads with a minimum length of 75 bases. The quality of each sequenced nucleotide was evaluated on adapter-filtered and quality-trimmed RNA-seq reads using the FASTX toolkit [22], and a mean Phred quality score of 40.22 ± 1.58 was obtained. After adapter and quality filtering, RNA-seq reads were mapped to Bowtie2 [23] indexes of the horse reference genome (assembly EquCab 3.0) using the splice-aware aligner Tophat2 [24]. Sample-specific transcriptomes were assembled using Cufflinks and merged with the reference genome to create a set of known and novel isoforms using Cuffmerge [25]. A total of 28,625 full-length transfrags were identified. Alignment statistics and base coverage were obtained with SAMtools [26]. Samples showed on average 74% of sequencing reads mapping to the reference genome. Total gene expression abundance was quantified for unique sequence reads using HTSeq [27]. Genes with less than 2 sequence read count abundance across all 40 samples were removed from further analysis to reduce the number of genes with low expression, leaving 14,133 gene transcripts for gene set enrichment and differential gene expression analyses. RNA sequence files have been deposited in the NCBI Sequence Read Archive BioProject Submission SUB12520338.

#### 2.6.3. RNA-Seq Count Normalization and Transformation

Only genes whose expression was estimated across all samples were retained for downstream analysis. The resulting gene counts were normalized using trimmed mean of M-values (TMM). Raw gene transcript abundances were transformed to approximate a normal distribution by calculating the log_2_ counts per million (CPM), which is the log_2_ of the raw counts and scale-normalized library size ratio. The mean-variance trend of gene transcripts was estimated and incorporated in the variance modeling of the DE analysis as precision weights to account for observational level and sample-specific parameters shared across genes [28,29]. The CPM and precision weights were computed using the Bioconductor R package Limma [28,29].

Differential gene expression (DE) was identified using the Limma analysis pipeline [28,30]. This pipeline fits a generalized linear model with a mean-variance trend incorporated into precision weights for individual normalized observations and empirical Bayes *t*-tests relative to a threshold (TREAT) [31], where the threshold used in this analysis was a fold change of 1.1. The gene-wise fixed effects model with *a* horses, *b* timepoints and *c* diets used to test DE (log_2_ fold change: log_2_ FC) at depletion or repletion compared to pre-exercise within diet can be represented as:

$$y_{ijk}=\mu +\alpha_{i}+\beta_{i\left(j\right)}+\gamma_{k\left(ij\right)}+e_{l\left(ijk\right)}\;\{\begin{array}{c} \begin{array}{c} i=1,\ldots ,a_{}\\ j=1,\ldots ,b_{} \end{array}\\ k=1,\ldots ,c_{}\\ l=1,\ldots ,n_{} \end{array}$$

where *y* is the CPM of a gene for horse *i* at timepoint *j* and diet *k*, *μ* is the overall mean, $\alpha_{i}$ represents the effect of the *i*th horse, $\beta_{i\left(j\right)}$ represents the effect of the *j*th timepoint nested within the *j*th horse, $\gamma_{k\left(ij\right)}$ represents the effect of the *k*th diet within the *i*th horse and *j*th diet, and $e_{l\left(ijk\right)}$ represents the random error term. Multiple test correction was performed for the 14,133 tested genes using a false discovery rate (FDR) less than 0.05. 

#### 2.6.4. DE of Genes Impacting GLUT4 Translocation and Transcription

Expression of genes encoding proteins that impact GLUT4 translocation or expression was extracted from the transcriptomic analysis. For GLUT4 translocation, this included adenosine monophosphate kinase (*PRKAA1*, *PRKAA2*, *PRKAB1*, *PRKAB2*, *PRKAG1*, *PRKAG2*, *PRKAG3*), RAB GTPase activating protein 1 (*RABGAP1*), member RAS oncogene family (*RAB13*), Cbl proto-oncogene B (*CBLB*), TBC1 domain family member 4 (*TBC1D4*), and TBC1 domain family member 1 (*TBC1D1*) [2,32,33]. Transcriptional activators of *GLUT4* gene expression included thyroid hormone receptor alpha (*THRA*), thyroid hormone receptor beta (*THRB*) PPARG coactivator 1 alpha (*PPARGC1A*), CCAAT enhancer binding protein alpha (*CEBPA*), myocyte enhancer factor 2A (*MEF2*)*, SLC2A4* regulator (*SLC2A4RG*)*,* myogenic differentiation 1 (*MyoD*)*,* KLF transcription factor 15 (*KLF15*), sterol regulatory element binding transcription factor 1 (*SREBF1*) and nuclear respiratory factor 1 (*NRF-1*) [34,35,36]. Finally, suppressors of *GLUT4* transcription included tumor necrosis factor (*TNF*)*,* peroxisome proliferator activated Receptor gamma (*PPARG*)*,* and peroxisome proliferator activated receptor alpha (*PPARA*) [36].

#### 2.6.5. DE of All Genes Encoding Glucose Transporters

Data was extracted from the transcriptomic analysis for *GLUT1* (*SLC2A1), GLUT2* (*SLC2A2), GLUT3* (SLC2A3), *GLUT4* (*SLC2A4*), *GLUT5* (*SLC2A25*), *GLUT6* (*SLC2A6*)*, GLUT8* (*SLC2A8*)*, GLUT9* (*SLC2A9*), *GLUT10* (*SLC2A10*), *GLUT11* (*SLC2A11*) and *GLUT12* (*SLC2A12*), comparing expression at depletion and repletion time points with pre-exercise expression for both the HS and the LS-HF diets.

#### 2.6.6. Impact of Diet on Glucose Transporter Transcript Expression

The expression of glucose transporters *GLUT1* through *GLUT12* was evaluated in the RNA-seq dataset, and their proportionate expression was calculated by dividing the CPM for each transporter by the total *GLUT* mRNA expression and conveyed as a percentage. 

## 3. Statistical Analysis

The glycemic indices expressed as AUC for all diets were compared by One-Way repeated measures ANOVA and Tukey’s test. Blood glucose concentrations during the incremental exercise test were compared between the HS and LS-HF diets using a repeated measures ANOVA with horse included as a random effect. Glycogen concentrations for all diets were normally distributed (Anderson-Darling test) and compared using a 2 Way repeated measures ANOVA with the horse included as a random effect and Dunnett’s multiple comparison testing. Significance was set at *p* < 0.05, and a trend as *p* < 0.01. 

Differential expression of *GLUT*s was determined as described in 2.6.3. For total *GLUT* expression expressed as counts per million reads, the effects of diet, time (depletion, 24 and 72 h repletion) and their interaction were evaluated for each expressed *GLUT* using a mixed model analysis with diet, time and time × diet as fixed effects and horse as a repeated effect. Multiple test correction was used for the nine *GLUT* ANOVA analyses, setting significance at *p* = 0.006 (*p* = 0.05/9 = 0.006). Data analysis was performed using GraphPad Prism version 9.4.1 (GraphPad Prism, San Diego, CA, USA).

## 4. Results

### 4.1. Glycemic Index and Horses

The glycemic indices of the diets relative to oats were Oats 100 ± 28, HS 134 ± 30, MS 92 ± 48, LS-HF 32 ± 13. There was no significant difference in glycemic index between HS, MS and oats. The LS-HF concentrate had a significantly lower (*p* < 0.05) glycemic index than the other concentrates. 

All horses consumed their concentrate daily throughout the trial and maintained their pre-exercise body weight. Mean (SD) body weights were Period 1, 550 ± 41 kg; Period 2, 556 ± 48 kg; Period 3, 554 ± 49.3 kg. One of the six horses was removed from the dataset because it did not complete the trial due to an episode of rhabdomyolysis on the HS diet.

### 4.2. Blood Glucose

Resting blood glucose concentrations prior to the incremental exercise test were similar between the HS (5.7 ± 0.38 mmol/L) and MS (5.8 ± 0.22 mmol/L, LS-HF (5.9 ± 0.24 mmol/L) diets and did not differ significantly during the exercise test (Figure S1).

### 4.3. Muscle Glycogen Concentrations

**Figure 2:**
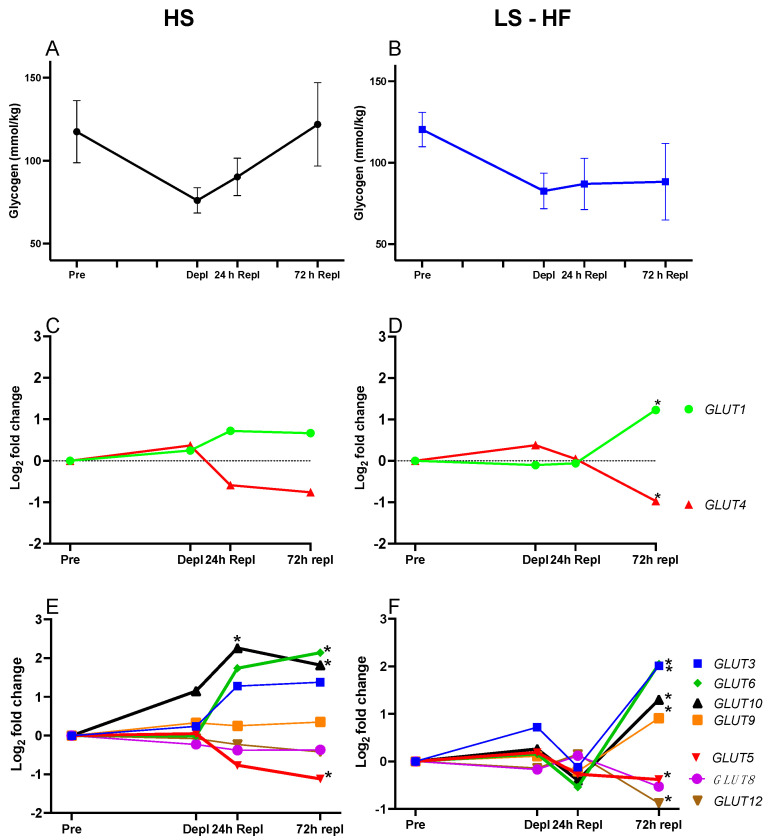
(**A**) Muscle glycogen concentrations in 5 horses prior to exercise, after 3 days of intense exercise (Depl) and 24 and 72 h after being fed the high-starch (HS) diet and rested. (**B**) Muscle glycogen concentrations after being fed the low-starch, high-fat (LS-HF)) diet. There was a significant effect of horse, time, and a time by glycogen interaction and trend (*p* = 0.07) for lower muscle glycogen concentrations on the LS-HF compared to the high-starch (HS) diet at 72 h repletion. (**C**) DE of *GLUT1* and *GLUT4* relative to pre-exercise expression identified using the Limma analysis pipeline on the HS diet. Notably, *GLUT4* did not increase in expression throughout repletion. (**D**) DE of *GLUT1* and *GLUT4* on the LS-HF diet. GLUT4 had a significant decrease in expression at 72h. (**E**) DE of other expressed glucose transporters on the HS diet. *GLUT6* and *GLUT10* had increased DE at 24 and 72 h. (**F**) DE of other expressed glucose transporters on the LS-HF diet. *GLUT3, GLUT6, GLUT9* and *GLUT10* had increased DE at 72 h. The delay in glycogen repletion on LS-HF coincided with delayed expression of *GLUT3*, *GLUT6* and *GLUT10* compared to HS. * Asterisks indicate significant DE relative to pre-exercise.

There was a significant effect of horse (*p* = 0.02) and time (*p* < 0.0001), and a significant time by diet interaction on glycogen concentrations (*p* = 0.03). Overall, glycogen concentrations were not significantly different between diets (*p* = 0.59); however, there was a trend (*p* < 0.1) toward lower glycogen concentrations at 72 h comparing LS-HF to both MS (Dunnett’s *p* = 0.08) and HS (*p* = 0.07) diets (Figure 2A,B and Figure S2). On average, from before to immediately after the third day of glycogen-depleting exercise, glycogen concentrations declined by 33 ± 17% on HS, 46 ± 20% on MS and 31 ± 8% on LS-HF. Glycogen concentrations increased linearly from depletion to 72 h repletion to near-resting glycogen concentrations; however, on LS-HF at 72 h repletion, mean muscle glycogen concentrations remained low. 

### 4.4. Transcriptomics

On average, 43 ± 8.1 million short-read pairs were sequenced per sample library. Adapter and quality filtering removed 26% of reads. The retained sequence reads were mapped to the EquCab 3.0 reference genome at 79% efficiency. Only the uniquely mapped reads were used to quantify transcript abundance (59% of total sequenced read pairs). The average depth of coverage per sequenced base was 63× with a 2% coverage of the reference genome. A total of 29,917 gene transcripts were expressed. After filtering for low count transcripts, 14,133 (47.2%) genes remained.

#### 4.4.1. Differential Expression Analyses

Out of the 14,133 genes identified in the transcriptomics data set, there were 240 differentially expressed genes (DEG) at depletion on the HS diet and 342 DEG on the LS-HF diet when compared to pre-exercise expression (Table S3). At 24 h repletion, there were 439 DEG on HS and, notably, zero DEG on LS-HF. At 72 h repletion, there were 1820 DEG on HS and 4010 DEG on LS-HF when compared to pre-exercise expression (Table S3). At each time point, the number of significantly upregulated DEG was greater than downregulated DEG (Table S3). Comparing expression of genes between HS and LS-HF at each time point, there were no significant DE genes analyzing the entire transcriptomic dataset.

#### 4.4.2. Differential Expression of Genes Impacting GLUT4 Translocation

**Figure 3:**
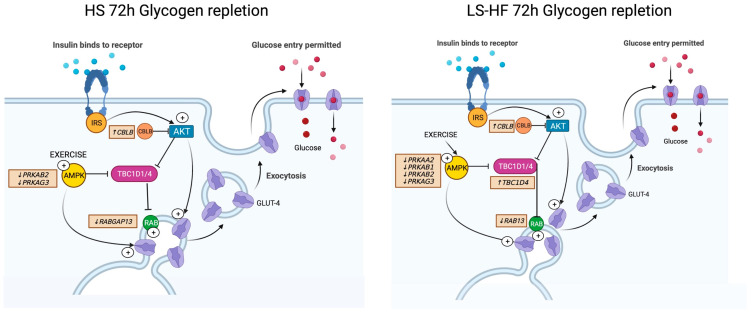
The change in expression of genes encoding proteins that impact GLUT4 translocation at 72 h of glycogen repletion compared to pre-exercise for horses on the high-starch (HS) (**left**) and low-starch, high-fat (LS-HF) (**right**) diets. Genes encoding several subunits of AMP kinase as well as RAB-signaling proteins had decreased expression, and the inhibitor *TBC1D4* (AS160) had increased expression on LS-HF, which combined could decrease GLUT4 translocation. *TBC1D1* was not expressed in the muscle samples.

After the glycogen-depleting exercise, there was no change in expression of genes encoding proteins that impact GLUT4 translocation for depletion and 24 h repletion time points on the HS diet (Table S4). At 72 h of repletion, when glycogen was near pre-exercise concentrations, translocation enhancers *RABGAP13* (−0.46, *adj p* = 0.009), *PRKAB2* (−0.66 log_2_FC *adj p* = 0.0247) and *PRKAG3* (−1.24 log_2_FC *adj p* = 0.0333) had significantly ↓DE whereas translocation enhancer *CBLB* had significantly increased (↑) DE (0.80 log_2_FC, *p* = 0.0038) on the HS diet (Figure 3, Table S4). 

In contrast, on the LS-HF diet, at depletion there was ↑DE of two subunits of the AMP-activated protein kinase (AMPK) that encodes proteins that induce GLUT4 translocation (*PRKAB1* 0.55 log_2_FC *adj p* = 0.0077and *PRKAG2* 1.06 og_2_FC *adj p* = 0.0034). At 24 h repletion on LS-HF, there were no DE genes impacting GLUT4 translocation (Table S4). However, when glycogen concentrations were still low at 72 h on the LS-HF diet, GLUT4 translocation suppressor *TBC1D4* (0.59 log_2_FC, *adj p* = 0.040) had significantly ↑DE and translocation enhancers had significantly ↓DE, including *RAB13* (−0.41, *adj p* = 0.0128)*, PRKAA2* (−0.87 log_2_FC *adj p* = 0.0097), *PRKAB1* (0.45 log_2_FC *adj p* = 0.0333), *PRKAB2* (−0.62 log_2_FC *adj p* = 0.0155) and *PRKAG3* (−1.30 log_2_FC *adj p* = 0.0126) (Figure 3, Table S4). GLUT4 translocation enhancer *CBLB* had ↑DE (0.85 log_2_FC, *adj p* = 0.011) at this timepoint.

#### 4.4.3. Differential Expression of Genes Impacting GLUT4 Transcriptional Activation or Repression

*GLUT4* expression relative to pre-exercise did not change significantly over time on the HS diet (Figure 2C), and none of the investigated activators of *GLUT4* expression were DE at depletion or 24 h repletion on the HS diet including *THRA*, *THRB*, *PPARGC1A*, *CEBPA*, *MEF2,SLC2A4RG, MyoD, KLF15*, *SREBF1* and *NRF-1* (Table S5) [34,35,36]. At 72 h repletion on HS, when glycogen concentrations were close to pre-exercise concentrations, decreased (↓)DE relative to pre-exercise of the *GLUT4* transcriptional activator *THRB* (−1.06 log_2_FC, *adj p* = 0.038) and the repressor *PPARA* (−0.76 log_2_FC, *adj p* = 0.048) was observed (Table S5).

**Figure 4:**
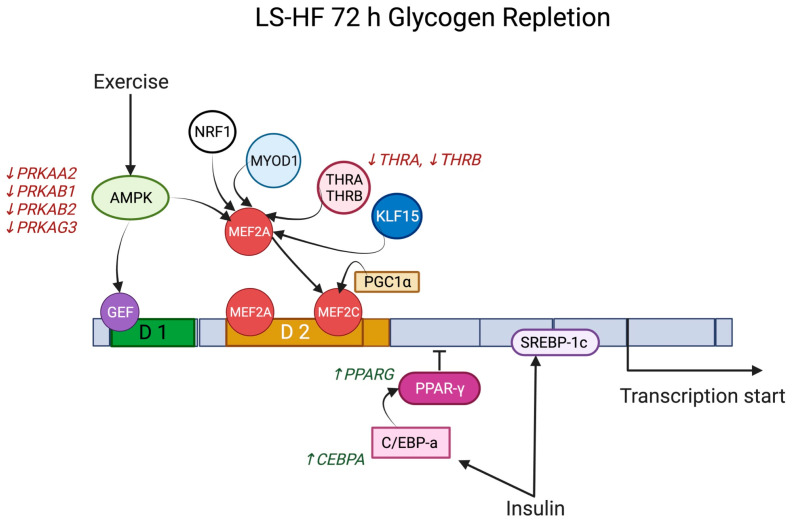
Differential expression of genes encoding activators or repressors of GLUT4 transcription at 72 h repletion on the low-starch, high-fat (LS-HF) diet. *GLUT4* transcriptional activators including four *PRKA* subunits of AMPK, as well as *THRA* and *THRB*, had significantly decreased expression (red) relative to pre-exercise. The *PPARG* gene encoding the *GLUT4* transcriptional suppressor peroxisome proliferator-activated receptor gamma and its activator *CEBPA* both had significantly increased expression. The net effect would be to decrease GLUT4 transcription even in the face of low muscle glycogen concentrations on the LS-HF diet at 72 h after exercise.

On the LS-HF diet, there was significantly ↓DE of *GLUT4* at 72 h repletion (−0.97 log_2_FC, *adj p* = 0.026) despite low muscle glycogen concentrations at this timepoint (Table S5). At depletion, but not at other timepoints, *GLUT4* transcriptional activator *PPARGC1A* (2.29 log_2_FC, *adj p* = 0.004) had significantly ↑DE on LS-HF (Table S5). At 72 h repletion, *GLUT4* transcriptional activators *THRA* (−0.62 log_2_FC, *adj p* = 0.026) and *THRB* (−0.94 log_2_FC, *adj p* = 0.038) had significantly ↓DE relative to pre-exercise and there was significantly ↑DE of the *GLUT4* transcriptional suppressor *PPARG* (1.03 log_2_FC, *adj p* = 0.004) on LS-HF (Figure 4, Table S5). Significantly, ↑DE of *CEBPA* (1.56 log_2_FC, *adj p* = 0.001), which activates the *SLC2A4* suppressor *PPARG* at 72 h repletion, was also found on LS-HF (Figure 4, Table S5).

The following genes impacting transcription or translocation were not expressed in the equine muscle samples: *EXOC7*, F*OXO1*, *HIF1A*, *PRKAA1*, *PRKAG1*, *TNF* (*TNFSF2*), *MEF2C*, *PPARD*, *PPARGC1B*, *TBC1D.*

#### 4.4.4. Differential Expression of Other Glucose Transporters

GLUT2 was not expressed in the muscle transcriptome. None of the 11 identified *GLUT* transcripts were DE at the depletion timepoint relative to pre-exercise (Figure 2C–F, Table S6). On HS, *GLUT10* had significantly ↑DE at 24 h repletion relative to pre-exercise, and at 72 h repletion *GLUT6* and *GLUT10* had ↑DE with ↓DE of *GLUT5* (Figure 2E, Table S6). On LS-HF, there were no DE *GLUT* until 72 h repletion, at which point *GLUT1*, *GLUT3*, *GLUT6*, *GLUT9*, *GLUT10* had ↑DE and *GLUT5* and *GLUT12* ↓DE relative to pre-exercise (Figure 2D,F, Table S6).

#### 4.4.5. Glucose Transporter Expression

**Figure 5:**
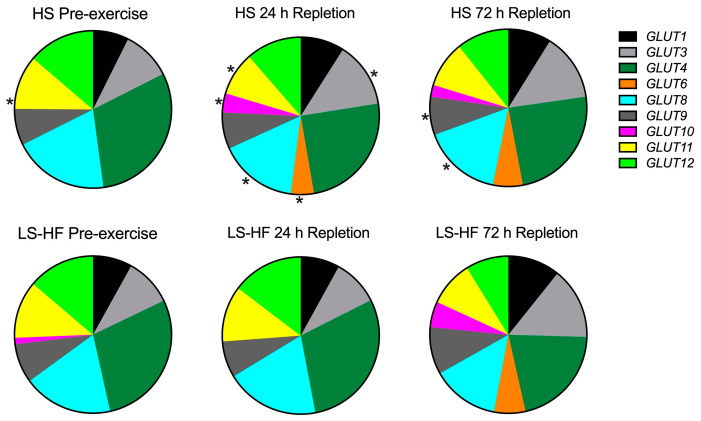
The proportionate expression of glucose transporters (CPM/total GLUT CPM) in muscle samples before exercise, after 24 and 72 h of repletion on the high-starch (HS) and low-starch, high-fat (LS-HF) diets. Asterisk indicates significant difference in expression (CPM) between diets (*p* < 0.006). Expression of *GLUT6* and *GLUT10* became apparent by 24 h on HS and 72 h on LS-HF.

Out of total *GLUT* expression, *GLUT4* was predominantly expressed, comprising 22–33% of glucose transporter mRNA across time points (Figure 5, Table S7). Within the *GLUT* subset, there was a significant effect of time for all expressed glucose transporters (*p* < 0.004). *GLUT4* expression decreased significantly (*p* < 0.001) during repletion, did not differ between diets (*p* = 0.372) and showed a significant time by diet interaction (*p* = 0.008) (Figure 5, Table S7). The second most abundant transcript in pre-exercise samples was *GLUT8* followed in order by *GLUT12*, *GLUT11*, *GLUT3*, *GLUT1* and *GLUT9* (Figure 5, Table S7). There was a significant time by diet interaction for *GLUT3* (*p* < 0.0001), *GLUT6* (*p* < 0.0001), *GLUT8* (*p* < 0.0001), *GLUT9* (*p* < 0.0001) and *GLUT10* (*p* < 0.0001) (Figure 5). *GLUT6* and *GLUT10* expression became evident at 24 h repletion on HS comprising 9% of all *GLUT* transcripts at 24 h, and 26% at 72 h repletion (Figure 5, Table S7). GLUT6 and GLUT10 expressions were delayed until 72 h repletion on LS-HF, at which point they comprised 21% of all expressed *GLUT* (Figure 5, Table S7). 

Within the GLUT data subset, there was lower expression of *GLUT10* (*p* = 0.0004) and *GLUT11* (*p* = 0.0005) before exercise on HS versus the LS-HF diet (Figure 5, Table S7). At depletion, there was no difference in *GLUT* expression between diets (Table S7). At 24 h repletion, there was greater expression of *GLUT3* (*p* < 0.0001), *GLUT6* (*p* =< 0.0001) and *GLUT10* (*p* = 0.0044) and lower expression of *GLUT8* (*p* = 0.0003) and *GLUT11* (*p* = 0.0005) on the HS versus LS-HF diet (Figure 5, Table S7). At 72 h repletion, lower expression of *GLUT9* (*p* = 0.001) and greater expression of *GLUT8* (*p* = 0.0004) were apparent on the HS versus LS-HF diet (Figure 5, Table S7). 

## 5. Discussion

**Figure 6:**
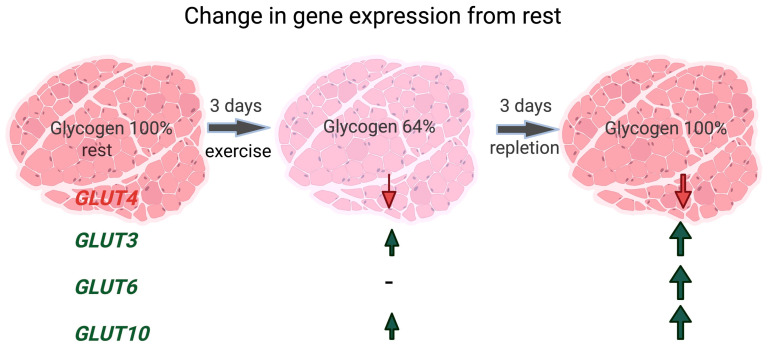
Summary of the changes in expression of genes encoding selected skeletal muscle glucose transporters from pre-exercise to post-exercise to 72 h of glycogen repletion. After 3 days of glycogen-depleting exercise, there was a mean 36% decline in muscle glycogen concentrations with gradual glycogen repletion over 72 h. No increase in activators of *GLUT4* translocation or *GLUT4* transcription occurred after glycogen depletion or repletion and *GLUT4* expression decreased with exercise. In contrast, with 72 h of repletion, *GLUT3*, *GLUT6* and *GLUT10* showed substantial increases in expression, as indicated by the size of the arrows.

Typical of equine muscle following intense exercise, the rate of glycogen repletion was slow, particularly in horses fed the LS-HF diet (Figure 2). Similar to other species, *GLUT4* was the primary facilitative glucose transporter expressed in equine skeletal muscle at rest (≈30% of total *GLUT* expression). However, unlike other species, GLUT4 expression did not change with glycogen-depleting exercise, and in fact decreased during repletion on LS-HF [5,6] (Figure 2). There were few molecular alterations to enhance GLUT4 expression. Only 1 of 13 genes encoding proteins that promote *GLUT4* transcription had increased DE at any timepoint (*PPARGC1A* at depletion on LS-HF) in our study. Further, only at the depletion timepoint on the LS-HF diet did two of 11 genes impacting GLUT4 translocation (two subunits of AMPK) have ↑DE (Figure 3). These results support a rate limiting role of GLUT4 in glucose transport in equine skeletal muscle. The most remarkable and novel finding in our study was that while there was little to no expression of *GLUT3*, *GLUT6* and *GLUT10* at rest, *GLUT3*, *GLUT6* and *GLUT10* comprised ≈25% of total *GLUT* expression after 24–72 h of repletion (Figure 5 and Figure 6). Thus, in the face of little change in *GLUT4* expression or likely translocation, equine muscle increases *GLUT3*, *GLUT6* and *GLUT10* expression during glycogen repletion potentially to enhance glucose transport. This mirrors GLUT3, GLUT6 and GLUT10 protein expression after resistance exercise in GLUT4-null mice [37,38]. The low-starch, fat-supplemented diet delayed muscle glycogen repletion and significant expression of *GLUT3*, *GLUT6* and *GLUT10* from 24 h on HS to 72 h on LS-HF. Thus, horses have a novel mechanism to regulate glucose transport and glycogen repletion, which is substantially altered by diet.

### 5.1. GLUT4 Translocation

In the absence of insulin or exercise stimulation, more than 95% of GLUT4 resides within intracellular membrane pools as a result of complex trafficking between the endosomal, trans-Golgi network and cycling pools (Figure 3) [32]. In most species, translocation of GLUT4 to the cell membrane occurs from signaling events initiated by AMPK with exercise or a PI3-K–dependent signal transduction network following binding of insulin to its receptor (Figure 3) [2]. In most species, negative regulators of translocation include TBC1D4 (AS160) and TBC1D1, which in turn regulate the Rab8A and Rab14 GTPases [32,33] and inhibit GLUT4 translocation and glucose uptake (Figure 3) [32]. Significant DE of only one gene that encodes initiators of GLUT4 translocation, one AMPK subunit, was observed after 3 days of intense exercise, and only in horses consuming the LS-HF diet. By 24 h of repletion, when glycogen concentrations were still low, no genes encoding promotors of GLUT4 translocation were DE on either diet. By 72 h repletion, expression of genes encoding subunits of AMPK primarily had decreased expression, as did expression of genes encoding GTPases on both diets (Figure 3). Thus, there appeared to be little impetus for increasing transcription of genes encoding activators of GLUT4 translocation in equine muscles during glycogen repletion despite low muscle glycogen concentrations. Our study did not examine the signaling cascade that directly impacts GLUT4 translocation. Nevertheless, our study provides evidence that equine muscle has a very limited transcriptional response to promote proteins involved in GLUT4 translocation. A lack of stimulus to enhance expression of genes promoting GLUT4 translocation is supported by a previous equine study that showed that only 15% of GLUT4 translocates to the cell membrane after insulin stimulation in equids, in contrast to other species [13].

### 5.2. GLUT4 Expression

A transient increase in *GLUT4* gene expression has been documented in humans, mice and rats after an exercise bout and is believed to be an early adaptive response to exercise [7,39,40]. Over time, the cumulative effect of short-term increases in *GLUT4* mRNA is to stimulate GLUT4 protein synthesis and enhance glucose transport [7,41]. In the present study, *GLUT4* expression did not increase after prolonged intense exercise, which concurs with a lack of increase in GLUT4 protein content after exercise in other equine studies [10,18]. The precise timing of changes in *GLUT4* expression vary among studies. In humans, *GLUT4* expression increases immediately and 3 h after an exercise session [42]. In rats, *GLUT4* mRNA increases immediately after exercise, but declines rapidly to baseline at 1.5 and 5 h after exercise [7]. In another study of exercise training in rats, *GLUT4* expression increased 24 h after the last bout of exercise and returned to baseline 48 h later [43]. Because muscle samples in our study were obtained 24 h after the preceding days’ exercise and immediately after the current days exercise, if changes occurred in *GLUT4* expression, we believe we would have detected them. However, no changes in *GLUT4* expression were identified at depletion in our study and *GLUT4* expression declined significantly at 72 h of repletion on the LS-HF diet in the face of low glycogen stores (Figure 2). Our results agree with a previous equine study that found no detectable change in muscle *GLUT4* mRNA content 4, 8, and 24 h after a single bout of submaximal exercise that produced 30% muscle glycogen depletion [44]. Furthermore, our findings agree with several previous equine studies that found no change in GLUT4 protein content following glycogen-depleting exercise in horses [10,44,45]. In fact, following 50% glycogen depletion, GLUT4 protein content did not change for up to 48 h after exercise in horses, regardless of dietary starch content, and was significantly reduced 72 h after exercise on HS, reflecting our findings of *GLUT4* gene expression [10]. 

*GLUT4* gene expression is controlled at the transcriptional level and initiated by the binding of specific proteins to two domains near the transcription initiation site [34]. One domain is the binding site for GEF encoded by *SLC2A4RG* and the other is a MEF2 binding site (Figure 4) [46,47]. Both domains and their associated binding factors are necessary for full GLUT4 expression in skeletal muscle [2]. With exercise, AMPK-mediated GLUT4 transcription involves increases in both GEF and MEF2 binding to the GLUT4 promoter (Figure 4) [36]. Consistent with a lack of increase in *GLUT4* expression, we did not find DE of *MEF2a, MEF2c, MEF2d* or *SLC2ARG* in equine gluteal muscle at any time point in our study. PPAR γ coactivator 1α (PGC-1α), which binds and activates *MEF2C*, was the only transcription factor that had increased DE (*PPArGC1A*) at depletion, but only on LS-HF not the HS diet [35]. An increase in expression of the gene encoding the AMPK β1subunit (*PRKAB1*) was only found at 72 h repletion on the LS-HF diet (Figure 4). The other genes encoding DE α,β, and γ AMPK subunits all had significantly decreased DE at 72 h on both diets (Figure 4). MyoD and thyroid receptor-α function cooperatively with MEF2 to modulate GLUT4 expression and their encoding genes (*MYOD1* and *THRA*) were not DE on the HS diet and *THRA* had significantly decreased DE at 72 h repletion on the LS diet (Figure 4) [36]. Further, KLF15 acts in synergy with MEF2A to activate the GLUT4 promoter and was not a DE gene at any time point on either diet [36]. C/EBP-a (*CEBPA*) and SREBP-1c (*SREBF1*) are known to impact insulin-mediated GLUT4 gene expression, and only *CEBPA* had increased DE and only at the 72 h timepoint on LS-HF when GLUT4 had significantly decreased expression (Figure 4) [34]. NRF-1, a downstream target of PGC-1α, also increases GLUT4 expression and glucose transport capacity; however, *NRF-1* was not a DE gene at any time point on either diet [35]. Thus, apart from *PPARGC1A,* the results of our study did not find evidence of increased expression of genes encoding transcriptional activators of GLUT4, supporting previous studies that show no increase in GLUT4 gene or protein expression after exercise and during glycogen repletion in horses [10,44]. The gene expression results in the present study indirectly evaluate GLUT4 transcriptional activation and do not provide evidence of binding of the encoded transcription factors to the GLUT4 domains. However, if GLUT4 transcription is limited, as was the case in our study, potential compensatory increased expression of genes encoding transcription factors might be expected.

### 5.3. Limitations to Glycogen Repletion in Horses

Glucose uptake across the sarcolemma appears to be the rate-limiting step in glycogen repletion in the horse [10,11,18]. Limited sarcolemmal glucose transport appears to primarily be due to a lack of a substantial increase in GLUT4 expression and translocation in equine skeletal muscle in response to both exercise and insulin stimulation [13,18,43]. One proposed explanation for this limitation is that insulin-dependent signaling pathways regulating GLUT4 are already at a near maximal physiologic limit in horses [17,18]. However, exercise has an additive effect with insulin on GLUT4 translocation in other species, and in our study, exercise did not appear to enhance *GLUT4* expression, indicating that there is more to limited glucose transport than maximal insulin signaling in horses [40]. It has also been suggested that the relatively high resting muscle glycogen concentrations typical of the equine species may partially prevent further GLUT4 translocation to the plasma membrane via a negative feedback pathway [18]. However, at the point of 30% glycogen depletion achieved in our study, the low glycogen concentrations would not have impacted translocation and glucose uptake, making high glycogen concentrations in the horse an unlikely explanation for limited glucose uptake. Horses have a relatively short small intestine, which could limit absorption of sugar, as well as its availability to skeletal muscle and glycogen synthesis; however, this would not explain the lack of increase in GLUT4 expression. An alternative explanation for the horse’s unique limitation to muscle glucose transport could be a protective evolutionary adaptation that restricts muscle glucose transport. This is supported by the almost complete lack of activation of any genes enhancing GLUT4 expression or translocation. Several prevalent equine myopathies are negatively impacted by high dietary nonstructural carbohydrate diets, and laminitis is a potential severe complication of high-starch feeds [14,48,49,50,51]. Thus, selection pressure could have been away from rapid glucose absorption and transport and its potential negative impact on health. The potential negative impact of rapid glucose uptake on horse health should be considered before attempting to pharmacologically overcome slow glucose transport and glycogen resynthesis in horses. 

### 5.4. Expression of Other Glucose Transporter

Ours is the first study to comprehensively evaluate expression of all potential *GLUT* in equine muscle. The novel finding in our study was that after 24–72 h of glycogen repletion, the expression of *GLUT3, GLUT6* and *GLUT10* increased in horses, with the timing of the increase depending on the diet (Figure 2 and Figure 6). In resting muscle *GLUT3*, *GLUT6* and *GLUT10* comprised 5% (HS) to 11% (LS-HF) of total *GLUT* mRNA; however, at 24 h of repletion, *GLUT3, GLUT6* and *GLUT10* comprised 23% of total *GLUT* mRNA on the HS diet. A delay in expression of *GLUT3*, *GLUT6* and *GLUT10* occurred until 72 h repletion on the LS-HF diet, at which point they comprised 27% of total *GLUT* mRNA compared to 22% for *GLUT4* expression. It is possible that the increase in expression of *GLUT3*, *GLUT6* and *GLUT10* reflects an attempt to increase GLUT3, GLUT6 and GLUT10 protein expression to enhance muscle glucose transport in the absence of enhanced GLUT4 expression. 

The findings are notably similar to GLUT4-null mice. In the absence of GLUT4, fed GLUT4-null mice can replete muscle glycogen concentrations within 24 h of exercise [52]. Fed GLUT4-null mice are also able to increases muscle glucose transport 2-fold following exercise (swimming) [37,53]. The increase in glucose transport in GLUT4-null mice and the ability to replete glycogen are speculated to be due to activity of other GLUT transporters. Resistance training studies have recently been performed in GLUT4-null mice in which the plantaris muscle in one hindlimb is overloaded through a synergistic ablation surgery and glucose transporter expression is compared between the sham operated limb and the ablated limb [38]. A marked increase in expression of GLUT3, GLUT6 and GLUT10 occurred in the overloaded limb in conjunction with increased glucose transport, suggesting that one or more of GLUT3, GLUT6 and GLUT10 transporters is responsible for enhanced glucose transport in the absence of GLUT4 [38]. 

### 5.5. Effect of Diet

An official definition of a high- or low-starch horse feed has not been established by the Association of American Feed Control Officials (AAFCO). Kentucky Equine Research measured the starch content of 292 performance horse feeds manufactured in North America and Europe (Pagan unpublished). The median starch content of these feeds equaled 25.5% starch (100% DM basis) and the 10th and 90th percentile equaled 12.1% and 39.0%, respectively. Compared to these feeds, the starch content of the HS feed used in our study can be defined as high (56.9%) and starch content of the LS-HF feed can be defined as low (6.4%). 

When fed the HS diet, horses were able to gradually replete muscle glycogen concentrations to near resting levels within 72 h. However, it is clear from the present study that feeding a LS-HF diet after glycogen-depleting exercise resulted in a prolonged delay in restoring muscle glycogen concentrations, similar to findings in another low-starch (starch 4.3% of a hay diet, no added fat) glycogen repletion trial in horses [10]. This delay was mirrored in our study by a delay in the appearance of increased *GLUT3*, *GLUT6* and *GLUT10* expression. In other species, high-fat feeding has been shown to selectively impair insulin-stimulated, but not contraction-pathway-mediated, glucose transport by reducing GLUT4 translocation to the plasma membrane [52]. Further, in rat skeletal muscle, high-fat diets downregulate GLUT4 mRNA, possibly through the activation of PPARG [54,55]. PPARG had significantly ↑DE at 72 h of repletion on LS-HF, which corresponded to significantly ↓DE of GLUT4 in our study. The temporal delay in expression of *GLUT3*, *GLUT6* and *GLUT10* on LS-HF, in conjunction with significantly delayed glycogen repletion, further increases interest in studying the role of GLUT3, GLUT6 and GLUT10 in glucose uptake in equine skeletal muscle during glycogen repletion. 

The exercise protocol used in the present study was more intense than that is usually performed by many sport horses. Thus, for many sport horses, daily glycogen depletion is likely less than 30%, and a delay in glycogen repletion may not have an impact on performance. However, for horses competing in several days of strenuous exercise, such as three-day events or horses competing in 50–100-mile endurance races, nutritionists and horse owners should be conscious of the significant delay in glycogen repletion with LS-HF diets because these diets have become a mainstay of equine nutrition. 

## 6. Conclusions

In conclusion, muscle glycogen concentrations declined by 30% after 3 days of intense exercise on both the HS and LS-HF diets, with near complete glycogen repletion occurring after 72 h of repletion in horses on the HS, but not the LS-HF, diet. Following exercise, and during the 72-h glycogen repletion period, GLUT4 gene expression did not increase, and few genes impacting GLUT4 transcription or genes encoding proteins impacting GLUT4 translocation were differentially expressed relative to pre-exercise. Rather, a novel pattern of glucose transporter expression occurred during glycogen repletion, which included an increase in expression (relative to pre-exercise) of *GLUT10* after 24 h repletion on HS and increased expression of *GLUT3*, *GLUT6* and *GLUT10* after 72 h on both HS and LS-HF diets. Notably, the increase in expression of *GLUT3*, *GLUT6* and *GLUT10* resembles the pattern of GLUT protein expression that occurs in resistance-exercised GLUT4-null mice. Thus, the response of equine muscle to glycogen depletion and repletion bares a remarkable resemblance to resistance trained GLUT4 null mice. 

## Data Availability

The data that support the findings of this study will be openly available in NCBI Sequence Read Archive at Project Submission SUB12520338.

## Supplementary Materials

The following supporting information can be downloaded at: https://www.mdpi.com/article/10.3390/metabo13060718/s1. Table S1: Nutrient analysis of Diet Concentrates (LS = Low-starch; MS = Medium-starch; HS = High-starch) and Hay (Timothy Grass), on a DM basis. Table S2: Nutrient analysis of dietary concentrates high-starch (HS), medium-starch (MS) and low-starch, high-fat (LS-HF), as well as hay on a DM basis. Table S3: the number of differentially expressed (DE) genes in the total dataset for 3 time points, as well as the number that were upregulated or down-regulated relative to pre-exercise gene expression for horses on the high-starch (HS) and the low-starch, high-fat (LS-HF) diets. Table S4: Gluteal muscle differential expression relative to pre-exercise for genes encoding activators or suppressors of GLUT4 translocation in horses on a high-starch (HS) and low-starch, high-fat (LS-HF) diets. Data is presented for time points using glycogen depletion post-exercise, 24 h and 72 of repletion. Expression is presented as log_2_ fold change (FC). Adjusted *p* values represent an FDR of *p* < 0.05 and bolded asterisks indicates statistical significance. Table S5: Gluteal muscle differential expression relative to pre-exercise for genes encoding activators or suppressors of GLUT4 expression in horses on the high-starch (HS) and low-starch, high-fat (LS-HF) diets. Data is presented for time points, glycogen depletion post-exercise, 24 h and 72 of repletion. Expression is presented as log_2_ fold change (FC). Adjusted *p* values represent an FDR of *p* < 0.05 and bolded asterisks indicate statistical significance. Table S6: Differential expression of glucose transporters immediately after the third exercise session (depletion) and after 24h and 72 h of glycogen repletion in comparison to pre-exercise. Data are expressed as log2 fold change (FC) and with the adjusted (Adj) *p* values for an FDR of *p* < 0.05. Table S7: The gene expression of glucose transporters (counts per million reads CPM) in gluteal muscle samples obtained before exercise, after 3 days of glycogen-depleting exercise and 24 and 72 h after a period of repletion on either isocaloric high-starch or a low-starch, high-fat diet. Asterisk indicates significant difference from the corresponding timepoint on the LS-HF diet. Figure S1: Blood glucose concentrations before and at each speed of the incremental exercise test. There was no significant difference in glucose concentrations between the HS and LS-HF diets. Figure S2: Muscle glycogen concentrations in 5 horses prior to exercise, after 3 days of intense exercise, and 24 and 72 h after being fed their respective diets and rested. There was a significant effect of horse, time, and a time by glycogen interaction. Overall, glycogen concentrations did not differ between diets, but there was a significant time x diet interaction and trend (*p* < 0.08) for lower muscle glycogen concentrations on the low-starch, high-fat (LS-HF) compared to the medium-starch (MS) or high-starch (HS) diets.
