# Supplementary material for: Novel Expression of GLUT3, GLUT6 and GLUT10 in Equine Gluteal Muscle Following Glycogen-Depleting Exercise: Impact of Dietary Starch and Fat
Source: Metabolites. 2023 Jun 1;13(6):718. doi: 10.3390/metabo13060718 (PMC10301051; doi:10.3390/metabo13060718)
Supplement: Supplementary file 1 [file metabolites-13-00718-s001.zip › Supplemental Material.pdf]

Table S1. Nutrient analysis of Diet Concentrates (LS-HF=Low-starch; MS=Medium Starch; HS=High starch) and Hay (Timothy Grass), on a DM basis.

Nutrient		LS-HF	MS	HS
Moisture	%	10.7	9.8	10.4
Dry Matter	%	89.3	90.2	89.6
Crude Protein	%	9.4	10.9	11.0
Digestible Energy	Mcal/kg	3.1	3.1	3.0
ADF	%	25.3	29.0	40.5
NDF	%	37.1	41.9	57.1
Hemicellulose[1]	%	11.8	12.8	16.6
Starch	%	25.7	17.8	3.2
Sugars	%	10.0	9.6	8.9
NSC[2]	%	35.6	27.3	12.1
Crude Fat	%	3.8	5.1	8.0
Ash	%	5.7	6.0	6.8

[1]Hemicellulose: calculated as NDF-ADF; ADF= acid detergent fiber, NDF = neutral detergent fiber, [2]NSC= nonstructural carbohydrate calculated as Starch+Sugars

Table S2. Nutrient analysis of Diet Concentrates (LS-HF=Low-starch; MS=Medium Starch; HS=High starch) and Hay (Timothy Grass), on a DM basis.

Nutrient		LS-HF	MS	HS	HAY
Moisture	%	10.8	9.6	11.6	10.0
Dry Matter	%	89.2	90.4	88.4	90.0
Crude Protein	%	12.0	11.8	9.4	10.2
Digestible Energy	Mcal/kg	3.3	3.6	3.6	2.7
ADF	%	41.5	15.7	7.3	39.7
NDF	%	59.0	24.8	14.1	55.5
Hemicellulose[1]	%	17.5	9.1	6.8	15.8
NFC	%	14.7	53.9	69.6	26.2
Starch	%	6.4	39.1	56.9	0.7
Sugars	%	5.9	7.4	8.3	11.3
NSC[2]	%	12.3	46.5	65.2	12.0
Crude Fat	%	14.3	7.8	4.8	3.0
Ash	%	5.9	4.1	3.5	7.5

[1]Hemicellulose: calculated as NDF-ADF; [2]NSC: calculated as Starch+Sugars.
ADF=Acid Detergent Fiber; NDF=Neutral Detergent Fiber; NFC=Non-fibrous Carbohydrates; NSC=Non-structural carbohydrates.

Table S3. The number of differentially expressed (DE) genes in the total dataset for 3 time points as well as the number that were upregulated or down-regulated relative to pre-exercise gene expression for horses on the high starch (HS) and the low starch, high fat (LS-HF) diets.

HS compared to pre-exercise			
**Time point**	DE Genes	Up-regulated	Down-regulated
**Depletion**	240	146	94
**Repletion 24h**	439	295	144
**Repletion 72h**	1820	1027	793
**LS-HF compared to pre-exercise**			
**Time point**	DE Genes	Upregulated	Down-regulated
**Depletion**	342	209	133
**Repletion 24h**	0	0	0
**Repletion 72h**	4010	2458	1552

Table S4: Gluteal muscle differential expression relative to pre-exercise for genes encoding activators or suppressors of GLUT4 translocation and *GLUT4* expression in horses on a high starch (HS) and low starch high fat (LS-HF) diets . Data is presented for time points glycogen depletion post-exercise, 24 h and 72 of repletion. Expression is presented as $\log_2$ fold change (FC). Adjusted *P* values represent an FDR of $P<0.05$ and bolded asterisks indicates statistical significance.

HS						
Gene	Post Depletion		24 h Repletion		72 h Repletion	
	Log2 FC	Adj P	Log2 FC	Adj *P*	Log2 FC	Adj *P*
			Activators of GLUT4 translocation			
PRKAA1	0.18	8.42E-01	0.12	8.18E-01	0.11	8.11E-01
PRKAA2	-0.17	9.17E-01	-0.2	7.54E-01	-0.41	2.95E-01
PRKAB1	0.40	7.58E-02	0.23	4.66E-01	0.34	1.06E-01
PRKAB2	-0.27	6.40E-01	-0.54	1.31E-01	-0.66*	2.47E-02
PRKAG2	0.59	1.37E-01	-0.28	5.59E-01	-0.23	5.91E-01
PRKAG3	-0.67	4.08E-01	-0.51	5.18E-01	-1.24*	3.33E-02
RABGAP1	-0.11	9.83E-01	-0.25	4.11E-01	-0.28	2.19E-01
RAB13	0.27	3.92E-01	-0.34	1.75E-01	-0.41*	3.72E-02
CBLB	0.08	1.00E+00	0.34	5.12E-01	0.80*	3.38E-02
			Suppressors of GLUT4 translocation			
TBC1D4	-0.1	9.92E-01	-0.43	2.84E-01	-0.39	2.52E-01
LS-HF						
Gene	Post Depletion		24 h Repletion		72 h Repletion	
	Log2 FC	Adj P	Log2 FC	Adj *P*	Log2 FC	Adj *P*
			Activators of GLUT4 translocation			
PRKAA1	0.07	9.72E-01	-0.13	1.00E+00	0.27	
PRKAA2	-0.19	8.42E-01	0.2	1.00E+00	**-0.87***	9.79E-03
PRKAB1	**0.55***	7.74E-03	0.16	1.00E+00	**0.45***	1.28E-02
PRKAB2	-0.09	9.46E-01	0.17	1.00E+00	**-0.62***	1.55E-02
PRKAG2	**1.06***	3.40E-03	0.04	1.00E+00	-0.23	5.32E-01
PRKAG3	-0.76	3.09E-01	-0.19	1.00E+00	**-1.30***	1.26E-02
RABGAP1	-0.21	6.20E-01	-0.07	1.00E+00	**-0.46***	8.98E-03
RAB13	-0.19	6.85E-01	0.11	1.00E+00	-0.35	5.35E-02
CBLB	0.19	8.52E-01	-0.1	1.00E+00	**0.85***	1.10E-02
			Suppressors of GLUT4 translocation			
TBC1D4	-0.27	6.54E-01	-0.18	1.00E+00	-0.59	3.99E-02

Table S5: Gluteal muscle differential expression relative to pre-exercise for genes encoding *GLUT4* expression in horses on a high starch (HS) and LS-HF diet. Data is presented for time points glycogen depletion post-exercise, 24 h and 72 of repletion. Expression is presented as log_2 fold change (FC). Adjusted *P* values represent an FDR of $P<0.05$ and bolded asterisks indicates statistical significance.

HS						
GLUT4 transcriptional activators						
Gene	Post depletion		24 h Repletion		72 h Repletion	
	Log_2 FC	Adj P	Log_2 FC	Adj P	Log_2 FC	Adj P
THRA	-0.35	4.94E-01	-0.54	1.56E-01	-0.43	1.78E-01
THRB	-0.29	8.21E-01	-1.01	1.08E-01	**-1.06***	3.80E-02
PPARGC1A (PGC1)	1.78	1.84E-02	-1.10	1.95E-01	-0.78	2.58E-01
CEBPA	-0.06	1.00E+00	0.92	1.42E-01	0.85	9.93E-02
MEF2A	-0.07	1.00E+00	0.02	9.80E-01	-0.01	9.91E-01
SLC2A4RG (GEF)	-0.57	7.80E-02	-0.61	9.24E-02	-0.48	1.03E-01
SREBPF1	0.58	2.67E-01	0.06	9.47E-01	-0.16	7.86E-01
MYOD1	-0.65	6.92E-01	0.03	9.89E-01	-0.21	8.86E-01
KLF15	-0.28	8.93E-01	-0.50	5.65E-01	-0.46	5.05E-01
NRF-1	-0.21	7.08E-01	0.00	9.95E-01	-0.14	7.23E-01
GLUT4 transcriptional suppressors						
PPARG	0.34	6.86E-01	0.29	6.49E-01	0.39	4.01E-01
PPARA	-0.15	9.55E-01	-0.65	1.60E-01	**-0.76***	4.80E-02
NF1	0.01	1.00E+00	0.28	3.70E-01	0.18	5.82E-01
LS-HF						
Gene	Post Depletion		24 h Repletion		72 h Repletion	
	Log_2 FC	Adj P	Log_2 FC	Adj P	Log_2 FC	Adj P
GLUT4 transcriptional activators						
THRA	-0.53	1.70E-01	-0.22	1.00E+00	**-0.63***	2.64E-02
THRB	-0.35	7.03E-01	-0.25	1.00E+00	**-0.94***	3.78E-02
PPARGC1A (PGC1)	**2.29***	4.11E-03	0.45	1.00E+00	-0.93	1.31E-01
CEBPA	0.28	8.10E-01	0.30	1.00E+00	**1.56***	1.37E-03
MEF2A	-0.04	9.93E-01	-0.09	1.00E+00	-0.35	1.26E-01
SLC2A4RG (GEF)	-0.58	7.18E-02	-0.69	1.15E-01	-0.28	3.20E-01
SREBPF1	1.21	5.18E-03	0.19	1.00E+00	-0.18	6.88E-01
MYOD1	-0.99	3.72E-01	-0.91	9.30E-01	0.19	8.36E-01
KLF15	-0.87	2.71E-01	0.06	1.00E+00	-0.71	1.80E-01
NRF-1	0.02	9.99E-01	-0.06	1.00E+00	0.19	4.98E-01
GLUT4 transcriptional suppressors						

PPARG	0.22	8.18E-01	-0.02	1.00E+00	**1.03***	3.93E-03
PPARA	-0.45	4.19E-01	-0.16	1.00E+00	**-0.89***	1.18E-02
NF1	0.27	4.57E-01	0.17	1.00E+00	0.16	5.88E-01

Table S6. Differential expression of glucose transporters immediately after the third exercise session (depletion) and after 24h and 72 h of glycogen repletion in comparison to pre-exercise. Data are expressed as $\log_2$ fold change (FC) and with the adjusted (Adj) P values for an FDR of P<0.05.

	HS						LS-HF					
Gene	Depletion		24h Repletion		72h Depletion		Depletion		24h Repletion		72h Depletion	
	$\log_2$ FC	Adj *P*	$\log_2$ FC	Adj *P*	$\log_2$ FC	Adj *P*	$\log_2$ FC	Adj *P*	$\log_2$ FC	Adj *P*	$\log_2$ FC	Adj *P*
GLUT1	0.25	8.61E-01	0.72	1.97E-01	0.67	1.37E-01	-0.10	9.65E-01	-0.06	1.00E+00	1.23	2.50E-03*
GLUT3	0.24	9.57E-01	1.28	1.05E-01	1.38	3.60E-02	0.72	4.66E-01	-0.12	1.00E+00	2.01	1.88E-03*
GLUT4	0.37	7.11E-01	-0.59	3.49E-01	-0.76	1.13E-01	0.38	6.68E-01	0.05	1.00E+00	-0.97	2.64E-02*
GLUT5	0.05	1.00E+00	-0.77	2.08E-01	-1.12	2.97E-02*	0.19	8.81E-01	-0.27	1.00E+00	-0.38	4.18E-01
GLUT6	-0.01	1.00E+00	1.74	6.68E-02	2.14	1.23E-02*	0.16	9.69E-01	-0.54	1.00E+00	2.05	3.25E-03*
GLUT8	-0.23	6.62E-01	-0.38	2.06E-01	-0.37	1.31E-01	-0.17	7.96E-01	0.12	1.00E+00	-0.53	1.39E-02*
GLUT9	0.33	6.29E-01	0.25	6.72E-01	0.35	3.81E-01	0.11	9.35E-01	-0.21	1.00E+00	0.91	4.07E-03*
GLUT10	1.15	2.41E-01	2.26	2.82E-02*	1.82	2.02E-02*	0.26	8.93E-01	-0.40	1.00E+00	1.30	2.29E-02*
GLUT11	-0.19	8.44E-01	-0.25	5.97E-01	-0.10	8.51E-01	-0.58	1.07E-01	-0.15	1.00E+00	-0.20	5.41E-01
GLUT12	-0.08	1.00E+00	-0.23	7.14E-01	-0.42	2.94E-01	-0.14	8.98E-01	0.15	1.00E+00	-0.88	1.15E-02*

* significantly differentially expressed (DE). No genes were DE at 24 h repletion on the LS-HF diet

Table S7: The gene expression of glucose transporters (counts per million reads CPM) in gluteal muscle samples obtained before exercise, after 3 days of glycogen depleting exercise and 24 and 72 h after a period of repletion on either isocaloric high starch or a low starch high fat diet. Asterisk indicates significant difference from the corresponding timepoint on the LS-HF diet.

Gene	Pre	Depletion	24 h Repletion	72 h Repletion	Pooled SD	Pre	Depletion	24 h Repletion	72 h Repletion	Pooled SD
	HS					LS-HF				
GLUT1	1.88	2.13	2.60	2.55	0.32	2.13	2.03	2.07	3.36	0.21
GLUT3	2.59	2.83	3.87*	3.97	0.29	2.58	3.30	2.46	4.59	0.31
GLUT4	7.73	8.10	7.14	6.96	0.36	7.54	7.92	7.59	6.57	0.10
GLUT5	-0.50	-0.45	-1.27	-1.62	0.33	-0.63	-0.44	-0.90	-1.01	0.43
GLUT6	-0.38	-0.39*	1.37*	1.76	0.36	-0.03	0.14	-0.57	2.02	0.23
GLUT8	5.03	4.80	4.65*	4.66*	0.12	4.87	4.70	4.98	4.34	0.11
GLUT9	1.91	2.24	2.16	2.26*	0.16	2.13	2.25	1.93	3.04	0.19
GLUT10	-1.13*	0.02	1.13*	0.69	0.67	0.34	0.60	-0.06	1.65	0.33
GLUT11	2.83*	2.64	2.58*	2.74	0.09	3.12	2.54	2.97	2.92	0.20
GLUT12	3.50	3.42	3.28	3.08	0.47	3.63	3.49	3.78	2.75	0.24
Total	23.47	25.34	28.00	27.05	3.17	25.68	26.51	24.25	30.23	3.17

* = significant differences between HS and LS-HF diets for that specific time point and transporter.

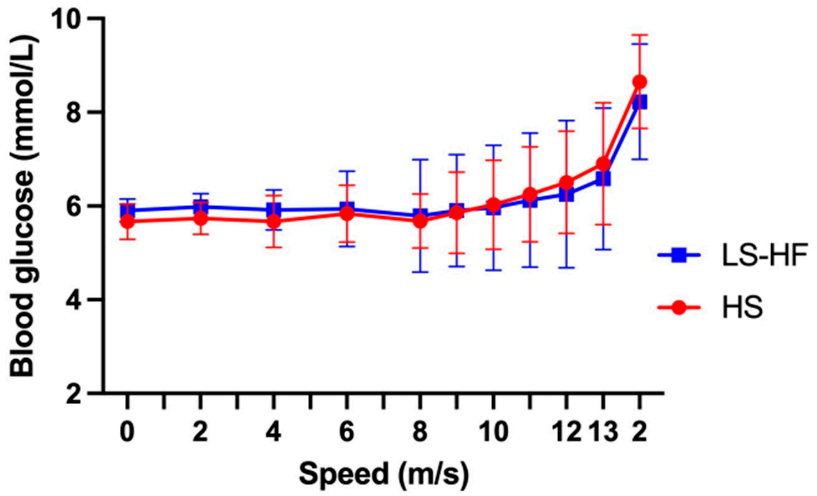

Figure S1. Blood glucose concentrations before and at each speed of the incremental exercise test. There was no significant difference in glucose concentrations between the HS and LS-HF diets.

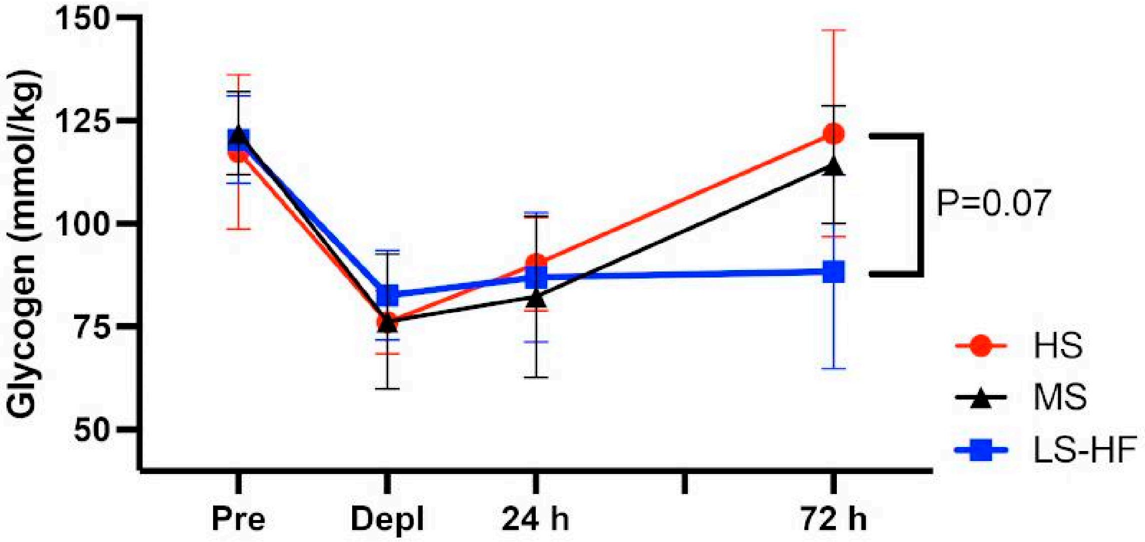

Figure S2. Muscle glycogen concentrations in 5 horses prior to exercise, after 3 days of intense exercise, and 24 and 72 h after being fed their respective diets and rested. There was a significant effect of horse, time, and a time by glycogen interaction. Overall, glycogen concentrations did not differ between diets, but there was a significant time x diet interaction and trend ($p < 0.08$) for lower muscle glycogen concentrations on the low-starch, high-fat (LS-HF) compared to the medium-starch (MS) or high-starch (HS) diets.
